# Application of Siamese Networks to the Recognition of the Drill Wear State Based on Images of Drilled Holes

**DOI:** 10.3390/s20236978

**Published:** 2020-12-06

**Authors:** Jarosław Kurek, Izabella Antoniuk, Bartosz Świderski, Albina Jegorowa, Michał Bukowski

**Affiliations:** 1 Institute of Information Technology, Warsaw University of Life Sciences, Nowoursynowska 159, 02-776 Warsaw, Poland; izabella_antoniuk@sggw.edu.pl (I.A.); Bartosz_Swiderski@sggw.edu.pl (B.S.); 2 Institute of Wood Sciences and Furniture, Warsaw University of Life Sciences, Nowoursynowska 159, 02-776 Warsaw, Poland; albina_jegorowa@sggw.edu.pl; 3 Department of Artificial Intelligence, Institute of Information Technology, Warsaw University of Technology, 02-776 Warsaw, Poland; michal.bukowski@buksoft.pl

**Keywords:** Siamese network, contrastive loss function, convolutional neural networks, deep learning, tool condition monitoring

## Abstract

In this article, a Siamese network is applied to the drill wear classification problem. For furniture companies, one of the main problems that occurs during the production process is finding the exact moment when the drill should be replaced. When the drill is not sharp enough, it can result in a poor quality product and therefore generate some financial loss for the company. In various approaches to this problem, usually, three classes are considered: green for a drill that is sharp, red for the opposite, and yellow for a tool that is suspected of being worn out, requiring additional evaluation by a human expert. In the above problem, it is especially important that the green and the red classes not be mistaken, since such errors have the highest probability of generating financial loss for the manufacturer. Most of the solutions analysing this problem are too complex, requiring specialized equipment, high financial investment, or both, without guaranteeing that the obtained results will be satisfactory. In the approach presented in this paper, images of drilled holes are used as the training data for the Siamese network. The presented solution is much simpler in terms of the data collection methodology, does not require a large financial investment for the initial equipment, and can accurately qualify drill wear based on the chosen input. It also takes into consideration additional manufacturer requirements, like no green-red misclassifications, that are usually omitted in existing solutions.

## 1. Introduction

Drill wear state recognition belongs to the larger group of problems called tool condition monitoring, which deals with the evaluation of different machine parts’ condition, as well as determining how long they can be used in the production process. Depending on the properties of each tool, as well as the requirements of the final product, different signals can be recorded and later tested using various methods, to obtain the final evaluation. Quite a few procedures in this direction also deal with the main topic of this paper, which is drill wear state recognition. From the manufacturer’s point of view, when the drill starts to become dull, it should be replaced as quickly as possible. Extending the use time of such a tool can result in poor product quality and therefore generate financial loss for the company. Manual evaluation of the drill state is possible and was initially done during the production process, but this is very time consuming, resulting in the prolongation of the entire procedure. A faster and more automated approach was needed, which resulted in extensive research on this subject. For example, one of the existing solutions focuses on measuring tool wear using two approaches: conventional methods and estimation with a customized software combining artificial neural networks and flank wear image recognition [1]. In this article, the authors noted that the automatic solution, while achieving slightly worse results, was still within the same range, and they presented some additional advantages, such as the lower cost.

Existing solutions vary greatly in their approach, especially the data collection methodology. As is often the case, especially when it comes to the usage of specialized equipment, the most visible advancements have been made in medicine. For example, in [2], the authors showed a miniaturized version of a device used for wireless intraoral force monitoring, which is capable of collecting and transmitting the required signals. In this field as well, image recognition and processing methodologies are used more often than in the wood industry. In [3], the authors automatically classified histological images using a histological ontology, and with the introduced improvements, they were able to recognize epithelial tissue, which was previously impossible. In the case of the wood industry, a significant number of solutions are sensor based, without including the possible advantages that the images of the checked element in the monitoring process can bring. As presented in [4], for a solution to find application in a commercial system, it usually needs to meet a series of different properties, dependent on the manufacturer’s requirements, while overall, solution complexity is not desirable. An additional solution requirement is an approach with the main focus on evaluating the tool state without disrupting the actual manufacturing process [5]. Originally, different sensors were used to measure such signals as feed force, noise, vibrations, acoustic emission, cutting torque, and others [6]. Such a solution, apart from the different devices used to collect the initial signals, also requires numerous preprocessing stages before obtaining samples that can be used. At each point, additional evaluation is required, to check if the chosen sensors are appropriate for the current work environment, if the registered signals are the correct ones, and if possibly the best diagnostic features are generated from them. Finally, from the initial set of features, a subset needs to be chosen for building the final classification model. What needs to be noted is that in the case of such solutions, not only the initial setup is complicated and costly from the manufacturer’s point of view, but additionally, an error at any key point can result in the final solution being unsatisfactory (in the case of choosing the wrong signal selection, apart from the resulting time loss, any costs for the required sensors should be treated as a financial loss). There are also a few different solutions that present an interesting approach to this problem. In [7], the authors used a large number of signals, extracted both from the signal and frequency domains, along with their wavelet coefficients, and evaluated them automatically to check how relevant they were to the presented problem based on the chosen set of properties. After the initial selection of the features used to estimate the tool wear, based on the final accuracy, the sensor and signal usability were evaluated. Similarly, in [8], various signals were registered over a wide range of cutting conditions, including such elements as thrust force and torque. In this case, the authors used a back-propagation neural network to predict the flank wear of a drill bit. In the solution presented in [9] as well, multiple sensors were used to collect the data, which were later integrated through a fuzzy logic model. Such a model is a combination of artificial neural networks and fuzzy logic and is capable of self-organization, self-adjustment, as well as learning from experience, and according to the authors, it was able to significantly increase the accuracy of tool wear estimation when compared to conventional approaches. In [10], the k-nearest neighbours algorithm was used in order to classify the drill condition into one of three initial classes. What is worth noting is that even while using diverse signals and different features, those solutions still obtained an accuracy below the 90% threshold. They also did not take into account the additional manufacturer requirements, such as minimizing the misclassification rate between the crucial red and green classes. The overall uncertainty of such solutions, when combined with the complicated setup and high initial costs, usually renders them inapplicable to real work environments.

The solution presented in this paper takes into account different approaches to deep learning in general (for elements such as the overall methods and applications [11], different architectures with AI applications as their main focus [12], or concerning deep learning in neural networks in general [13]), as well as the authors’ previous works on this subject [14,15,16,17], along with a few crucial observations that were made during that process. Firstly, instead of a set of specialized sensors, images of drilled holes are used as the input data for the presented algorithms. Since in that case, the only external piece of equipment required during the data collection process is a simple camera, the work needed at this stage is greatly minimized, and the initial costs are reduced. Different approaches to this subject were tested, using solutions based mainly on convolutional neural networks (CNNs; which do not require specialized diagnostic features and are currently considered some of top solutions when it comes to image recognition [18]). Initially, experiments were performed on limited set of data [14], with an additional approach checking the influence of the artificial data extension for the case with two classes [15]. Those first approaches proved to be quite promising, and in the following works, the data augmentation technique was combined with the transfer learning methodology [16]. In this case, the results were better than with much more complicated setups, using various sensors to collect the related signals, with the accuracy exceeding 93%. Another solution [17] this time incorporating classifier ensemble with different pretrained networks further improved the overall classification rate and obtained over 95% accuracy (including approaches attempting to beat the ImageNet large-scale visual recognition challenge [19], using solutions such as deep convolutional neural networks [20] or other pretrained networks, like AlexNet (available online) [21]).

While the above approaches showed that a high accuracy rate is possible even with a much simpler setup than the initial, sensor based solutions related to this subject, they still failed to incorporate the additional manufacturer requirement regarding the green-red misclassification rate. Usually, three classes are considered for this problem: the green class, describing a drill that is in good shape and that can be further used in the production process, the yellow class, for tools that are suspected of being worn, which requires additional evaluation by human expert, and finally, the red class, which describes the dull elements that should be replaced immediately, since their further use in the production process can result in poor product quality and financial loss. From the manufacturer’s point of view, a clear distinction between the red and green class is far more important than the other classifications. In the case of the yellow class in the standard approach, it will still be evaluated by a human expert, but if the green tool is classified as red, it will be discarded, while in the opposite case, a dull drill will be further used in the production process, resulting in holes with chipped edges. Enforcing this requirement has a higher impact on the overall solution adaptation to the actual work environment than the high overall accuracy.

The solution presented in this paper focuses on those aspects. The same type of input data are used as in the previous approaches, with only images of drilled holes being used as the training samples, without any additional signals. Furthermore, it is more focused on the green-red misclassification requirement. The Siamese network is used to build the main classifier, in order to ensure the best possible classification. This type of network was initially introduced for the face recognition problem and with CNN as its base and can be easily adjusted to drill wear state evaluation. The application of such a solution to the problem of drill wear classification was not previously studied, but the solution has obtained promising results. What is more, the presented method is highly adjustable to the changing parameters of the work environment, including changes to the samples’ characteristics.

This paper is organized as follows. The data collection methodology, as well as the obtained dataset are described in Section 2. Section 3 outlines the data preprocessing used to balance the dataset, as well as additional, required operations. Siamese networks in general, as well as the presented solution specifically are described in Section 4. Section 5 presents the results obtained during the experiments and a discussion about their quality in relation to previous solutions and possible areas for future work. Conclusions follow in Section 6.

## 2. Dataset

**Figure 1:**
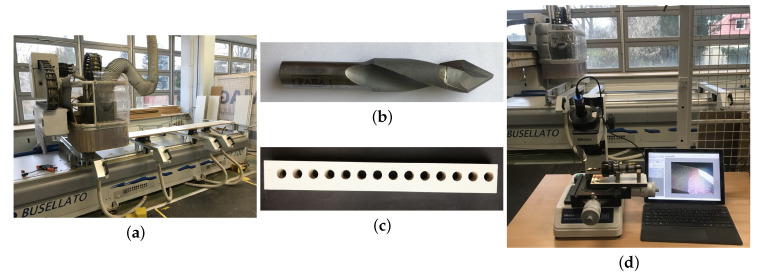
Equipment setup used during the experiments:Busellato Jet 100 machine centre (**a**), 12 mm Faba WP-01 drill (**b**), Kronopol U 511 SM melamine-faced chipboard sample (**c**) and workshop microscope TM–505, Mitutoyo (**d**).

The images used as the input for the presented algorithm were made using a Nikon D810 (Nikon Corporation, Shinagawa, Tokyo, Japan) single-lens reflex digital camera with a 35.9 × 24.0 mm CMOS image sensor. The entire process was performed in cooperation with the Institute of Wood Sciences and Furniture at Warsaw University of Life Sciences, Poland. For test purposes, a standard CNC vertical machine centre (Busellato Jet 100, Thiene, Italy) was used. Drilling was performed on a standard, melamine-faced chipboard (Kronopol U 511 SM; Swiss Krono Sp. z o. o., Żary, Poland), which is typically used in the furniture industry. The dimensions of the test piece were 300 × 35 × 18 mm. A regular, Faba WP-01 double-blade drill for through drilling (Baboszewo, Poland) equipped with a tungsten carbide tip was used. The drill’s overall length was 70 mm, with a shank length equal to 25 mm, a flute length of 40 mm, a shank diameter of 10 mm, and a 12 mm drill diameter. The clearance angle on the drill face was −15.45 degrees. The rake angle was equal to 0 degrees, and the helix angle for the tool used was 15 degrees. The images of the equipment used are presented in Figure 1.

The data set used in the current experiments was similar to that in the previous works [6,14,15,16,17], which was used both in the case of training the CNN network from scratch, as well as using transfer learning. It consisted of 5 image sets showing drilled holes, where each collection represented a separate tool. Samples were stored in the order in which they were made, showing the gradual wear of the drill and its effects on the hole edges. A total of 8526 images were taken, from which 3780 represented the green class, 2800 the yellow class, and 1946 the red class.

**Figure 2:**
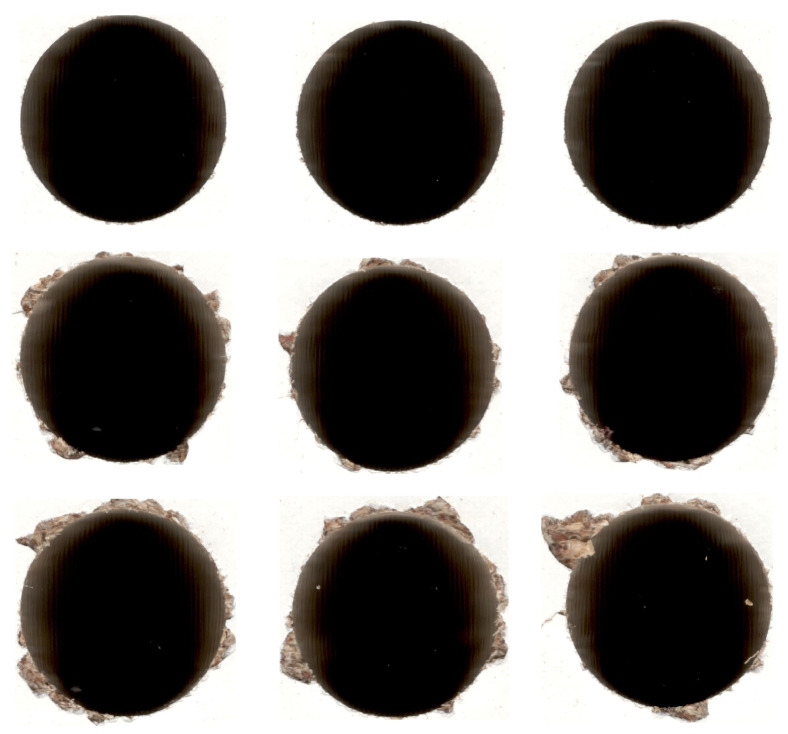
Example images representing holes made by drills with different wear classifications: green (**top**), yellow (**middle**), and red (**bottom**).

**Figure 3:**
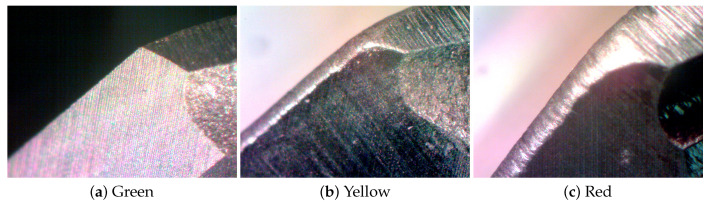
Images showing the wear of the outer drill corner for different classes: Green (**a**), Yellow (**b**) and Red (**c**). The microscope optical magnification equals 30×, while the total magnification for the monitor was 852.

Usually, three classes are used in drill wear state recognition: red, green, and yellow. In this case, the obtained samples were divided and labelled manually, using the drill wear rate. For the manual evaluation of the drill state, external corner wear (W(mm)) was adopted as the main condition indicator and was periodically monitored using a standard workshop microscope (TM–505; Mitutoyo, Kawasaki, Japan). Based on the obtained values, three classes for drill wear were selected: green for W < 0.2 mm, yellow for W in the range between 0.2 and 0.35 mm, and red for W > 0.35 mm. Those classes were also used for the drill wear definition in the current, automated approach. In the presented case, the yellow class was used mainly as a buffer for the manufacturer. In the case of the furniture industry, depending on the type of elements produced, different hole qualities can be acceptable. In this case, depending on the manufacturer’s preferences, the yellow class can later be assigned either to the green or red classes in the final production, hence expanding the overall method’s customizability. Example images representing different drill wear classes are presented in Figure 2. Images presenting drills at different wear stages are presented on Figure 3. Because of that division, there were some examples when the drill was reaching the yellow or red state, and hence, they were hard to classify; therefore, it was expected that especially with the additional requirements, the accuracy rate would not reach the 90% threshold.

## 3. Data Preprocessing

The original data set contained significantly more examples for the green class than the remaining two (yellow and red). Since CNN was used as a base network for the presented solution, it was not desirable for the data set to be imbalanced in such a way (it was important for the training process that each class be equally represented). To correct this, data augmentation methodologies were used, to ensure an even representation of each class (at this point, a simple rotation by 180 degrees was applied to the images, until the samples in each set reached a count equal to the best represented green class). Table 1 outlines the initial quantities for samples representing each class before and after data augmentation.

**Figure 4:**
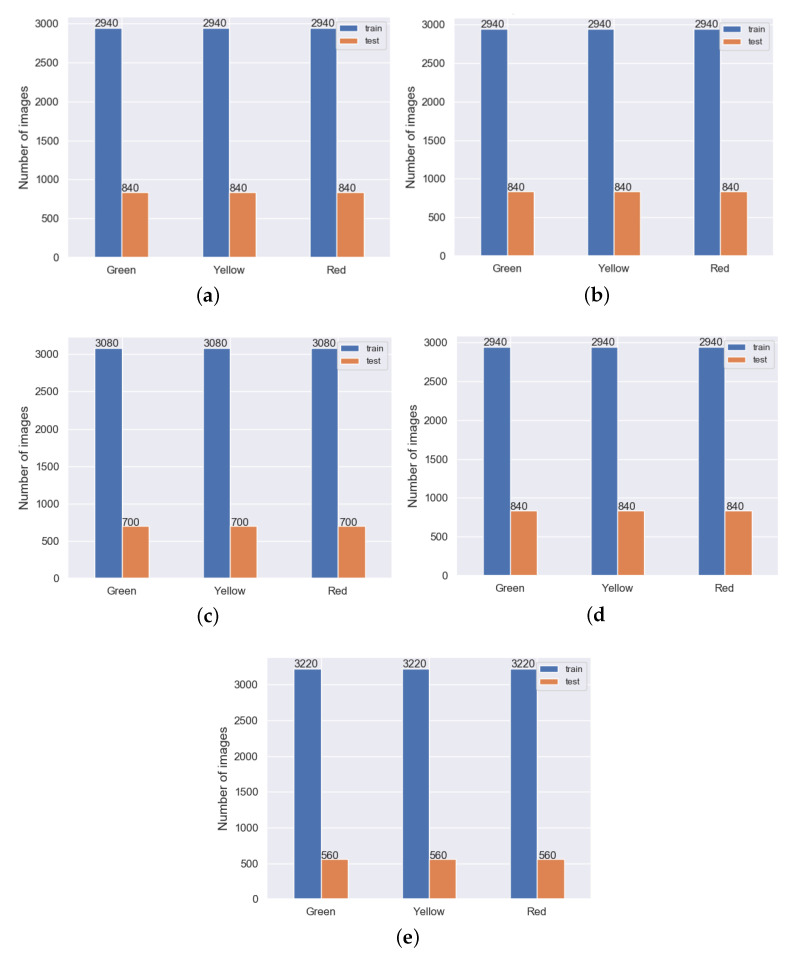
Fold data split during the training process using 5-fold cross-validation. The subsequent images represent the structure of: Fold 1 (**a**), Fold 2 (**b**), Fold 3 (**c**), Fold 4 (**d**), Fold 5 (**e**).

With the data balanced, the training process should not favour any of the classes. Initial operations performed on the data samples also included resizing each of the images on the fly to a size equal to 64 × 64 × 3 pixels. The training input was also normalized by dividing each value by 255, to ensure that they were in the (0, 1) range. Since 5-fold cross-validation was used, the input data were split between the 5 folds, and each of them was additionally divided into two subsets, the first for training and the second one for validation. The structure of each fold is shown in Figure 4.

## 4. Siamese Network for Drill Wear Classification

### 4.1. Siamese Network Architecture

Siamese networks are novel algorithms used in image recognition. The first approaches with this type of procedure focused specifically on face recognition. One of the first applications was a verification system for identifying workers in a building. When it comes to this problem in general, there are two main areas to consider: verification of whether a person in the current image is one stored in a database under a specified ID and recognizing if the person from the input image is one of those stored in the original database. Especially in systems with large amounts of users (i.e., gates used to restrict access to certain building parts), accuracy is a very important factor. While having a 99% recognition rate might be acceptable for other applications, it is not the case here. Even if such a system has a 1% error rate, with 100 people in the database, the possibility of not recognizing the current person correctly is still quite high. Additionally, for most cases with face recognition, the algorithm needs to be able to recognize the person while using a single image (the one-shot learning problem). Using CNN for such an approach is not good enough, since firstly, the amount of training data is minimal, and secondly, each time a new person is added to the system, the network would require retraining. This is where the approach used in Siamese networks has the advantage.

Firstly, for the face verification problem, instead of learning to recognize each person separately, the network learns a similarity function (or the difference between the image in the database and the currently presented image). In that case, if the difference between two images is greater than the set threshold, the person would be classified as different, and as the same in case of this value being below that threshold. In general, it can be described as:

(1) $$\begin{array}{c} \mathrm{If}\;x^{\left(i\right)}=x^{\left(j\right)}\;\mathrm{than}\;{∥f\left(x^{\left(i\right)}\right)-f\left(x^{\left(j\right)}\right)∥}^{2}\;\mathrm{is}\;\mathrm{small}\\ \mathrm{If}\;x^{\left(i\right)}\neq x^{\left(j\right)}\;\mathrm{than}\;{∥f\left(x^{\left(i\right)}\right)-f\left(x^{\left(j\right)}\right)∥}^{2}\;\mathrm{is}\;\mathrm{large} \end{array}$$

where:

- $x^{\left(i\right)}$, $x^{\left(j\right)}$: images representing sample from the database and the sample that is checked for similarity.
- f($x^{\left(i\right)}$), f($x^{\left(j\right)}$): functions describing each input image for distance measuring.

To be able to calculate this distance between the input images, both of them are encoded using identical CNN networks. They are then represented as feature vectors instead of the usual classification. In general, what is done at this point is that instead of using the final classifier from the CNN (or other network), the entire process stops at one of the embedding layers (or features derived from the original image). By using this approach, two different, comparable encodings of the images can be obtained, and the distance between them can be measured (i.e., using a dense layer, with 128 parameters as a vector used to compare the two images). The idea of first launching two identical CNN networks to produce feature vectors and secondly using those vectors to calculate the difference measure between images is the basis of the Siamese network architecture [22].

### 4.2. Contrastive Loss Function

The Siamese network is a good example of a solution that can distinguish between instances of different classes and specifically determine if the image that is provided as the input is the same as the one representing the original class. In terms of face recognition, it would determine if the same person is in the picture. In the case of the solution presented here, with some additional modifications, it should point to which drill wear class the provided example belongs.

To train networks used for such recognition, a few steps are required, as well as a definition of the function used to distinguish between positive and negative examples of each class. The presented solution needs to be able to do two things: recognize the same class in two different images and notice that the presented class is different than the one to which it is compared. To achieve that, the following images are required: the first one, containing the element representing a single class (called the anchor and denoted as A), the positive image example, containing the same class (positive, denoted as P), and the negative image, with a different class (negative, denoted as N). When the distance between those images is calculated (from the obtained image representation), the ideal outcome would produce results for which the distance between the images containing the element with the same class is lower than in the case of the images containing elements representing different classes. What is more important for this approach to work accurately is that this distance needs to be significant, reaching at least some predefined margin (i.e., if the difference between A and P is smaller by only 0.01 with regard to distance between A and N, it might not be enough). The above relation can be described using the following equations:

(2) $$\begin{array}{c} {∥f\left(x_{i}^{a}\right)-f\left(x_{i}^{p}\right)∥}_{2}^{2}+\alpha <{∥f\left(x_{i}^{a}\right)-f\left(x_{i}^{n}\right)∥}_{2}^{2}\\ \forall \left(f\left(x_{i}^{a}\right),f\left(x_{i}^{p}\right),f\left(x_{i}^{n}\right)\right)\in \tau \end{array}$$

where:

- α is a margin that is enforced between positive and negative pairs
- τ is the set of all possible triplets in the image set and has cardinality N.

At this point, the loss that needs to be minimized (generally described as the contrastive loss function [23]) has the form in Equation (3).

(3) $$L=\sum_{i}^{N}{\left[{∥f\left(x_{i}^{a}\right)-f\left(x_{i}^{p}\right)∥}_{2}^{2}-{∥f\left(x_{i}^{a}\right)-f\left(x_{i}^{n}\right)∥}_{2}^{2}+\alpha \right]}_{+}$$

**Figure 5:**
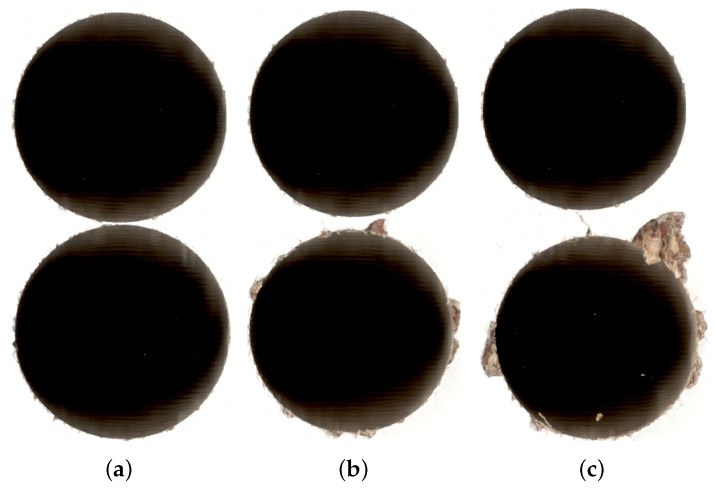
Examples of pairs used for training. The first pair - (**a**) - is a positive example, with label = 1; the following pairs are negative examples with label = 0, with green-yellow (**b**) and green-red (**c**) case. Anchors are presented at the top of each pair, while the checked image is at the bottom.

The margin parameter (α) is a hyperparameter that needs to be manually adjusted to each classification problem. In the case of the topic described in this paper, one type of positive pair (with label y = 1) and two types of negative pairs were used in that process (the first type containing a yellow class example and the second the red class, both with label y = 0). Figure 5 contains examples of each type of pair used for training. The method used during that process is presented in Algorithm 1 and was introduced in [24].
**Algorithm 1:** Siamese network training.Step 1: Generating the training set**for** each input sample $X_{i}$ **do** Using knowledge about the data set to find a set of samples such that each sample $X_{j}$ is similar to $X_{i}$. Pair sample $X_{i}$ with all the other training samples, and label pairs as $Y_{ij}=0$ if the samples are similar, $Y_{ij}=1$ otherwise**end for**Combine all the pairs to form the labelled training set.Step 2: Training**while** Convergence not reached **do** **for** each pair $\left(X_{i},X_{j}\right)$ **do**  if $Y_{ij}=0$, update W to decrease $D_{W}={∥f\left(x_{i}\right)-f\left(x_{j}\right)∥}^{2}$  if $Y_{ij}=1$, update W to increase $D_{W}={∥f\left(x_{i}\right)-f\left(x_{j}\right)∥}^{2}$ **end for****end while**

### 4.3. Siamese Network for Drill State Recognition

Since Siamese networks were originally for the face recognition problem, using a similar approach for the drill wear evaluation considered in this work, some adjustments were required. During the training process, first, the set of examples was created, where each example would contain two samples: anchor (A, to which the second sample will be compared, to determine if it is the same or different, corresponding to the picture of the person from the database in the face recognition problem) and either a positive (P) or a negative (N) example. In this case, instead of a single image that can either be the same or different (as with original application of this solution), a total of three classes were considered. First would be the positive example, with the same class as the anchor. In this case, two negative examples can be generated, containing either of the remaining classes.

The approach first divides the entire data set into positive and negative pairs. In the case of negative examples, to increase the diversity of the training set, the class is randomly chosen from the two that are different than the one to which the anchor belongs. Each of the initial images will generate two pairs used for training: one positive, where the anchor is paired with the image of the same class, and one negative, in which the anchor will be paired with an example from a different class. To calculate the distance between the images in consecutive samples, the contrastive loss function is used (see [24]).

**Figure 6:**
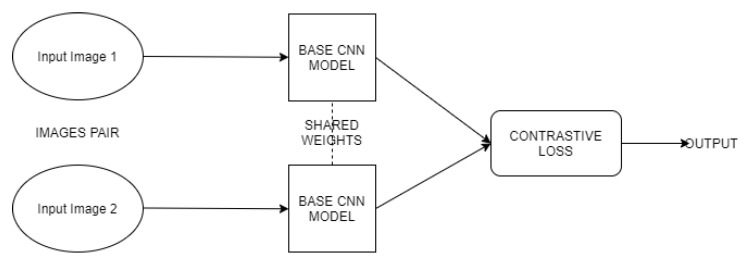
Outline of the Siamese network model used in the current experiments.

As a base network for the learning process, CNN is used, with three convolutional layers. The detailed structure of the model prepared for the problem chosen as a main topic of this paper is outlined in Listing 1. The Siamese network uses two identical CNN networks to generate the parameters for each of the images. The general structure of such a network is presented in Figure 6. The full algorithm that was applied to drill wear recognition is presented in Algorithm 2.
**Algorithm 2:** Network training algorithm used for the drill wear recognition problem.Create positive and negative pairs:**for** each of initial D = 3 classes **do** **for** all images in each class **do**  A = current image with index = i  P = next image from the same class with index = i + 1  PairPositive = (A, P) with label = 1  Randomly choose one of the remaining classes (different than the current one)  Choose negative image N with index = i  PairNegative = (A, N) with label = 0 **end for****end for**Calculate the contrastive loss function:**for** each created pair **do** Contrastive loss = $\frac{1}{m}\sum_{\left(i,j)\right)}^{m/2}y_{i,j}D_{i,j}^{2}+\left(1-y_{i,j}\right){\left[\alpha -D_{i,j}\right]}_{+}^{2}$**end for**Return classification

Listing 1 **List of layers used in the CNN model.**

Model: “CNN”
_________________________________________________________________
Layer (type) Output Shape Param #
=================================================================
conv2d_1 (Conv2D) (None, 62, 62, 64) 1792
_________________________________________________________________
activation_1 (Activation) (None, 62, 62, 64) 0
_________________________________________________________________
max_pooling2d_1 (MaxPooling2 (None, 31, 31, 64) 0
_________________________________________________________________
dropout_1 (Dropout) (None, 31, 31, 64) 0
_________________________________________________________________
conv2d_2 (Conv2D) (None, 29, 29, 64) 36,928
_________________________________________________________________
activation_2 (Activation) (None, 29, 29, 64) 0
_________________________________________________________________
max_pooling2d_2 (MaxPooling2 (None, 14, 14, 64) 0
_________________________________________________________________
dropout_2 (Dropout) (None, 14, 14, 64) 0
_________________________________________________________________
conv2d_3 (Conv2D) (None, 12, 12, 32) 18,464
_________________________________________________________________
activation_3 (Activation) (None, 12, 12, 32) 0
_________________________________________________________________
max_pooling2d_3 (MaxPooling2 (None, 6, 6, 32) 0
_________________________________________________________________
dropout_3 (Dropout) (None, 6, 6, 32) 0
_________________________________________________________________
flatten_1 (Flatten) (None, 1152) 0
_________________________________________________________________
dense_1 (Dense) (None, 128) 147,584
_________________________________________________________________
dropout_4 (Dropout) (None, 128) 0
_________________________________________________________________
dense_2 (Dense) (None, 50) 6450
=================================================================
Total params: 211,218
Trainable params: 211,218
Non trainable params: 0
_________________________________________________________________

## 5. Results and Discussion

During previous experiments [14,15,16,17], different approaches were tested and evaluated. While the obtained results were high in terms of overall accuracy, the entire solution still required improvement, when it comes to additional manufacturer requirements for the desired output. The main parameter concerned the number of errors between the red and green classes, which needed to be minimized. In this case, even a high overall accuracy would not be enough to compensate for this defect.

In the current approach, the Siamese network, adjusted to the presented classification problem, was used. To evaluate the obtained results, additional algorithms were implemented, choosing procedures that were successful in previous experiments, but similarly adjusting them to include the new requirement of minimizing the number of misclassifications between the red and green classes.

During the experiments, five-fold cross-validation was used (for the exact fold structure, see Figure 4). All experiments were performed using two NVIDIA TITAN RTX graphics card (with a total 48 GB of RAM), using the TensorFlow platform with the Python and Keras library, applying the Adam optimizer. Additionally, for the accuracy measurements, a custom function was used, defined as shown in Algorithm 3.
**Algorithm 3:** Accuracy function used for algorithm evaluation.** Require:** (y_true, y_predicted)  Compute classification accuracy with a fixed threshold on image distances  Return K.mean((K.equal(y_true, K.cast(y_pred < 0.5, y_true.dtype)))  model.compile(loss = contrastive_loss, optimizer = ‘adam’, metrics = [accuracy])

**Figure 7:**
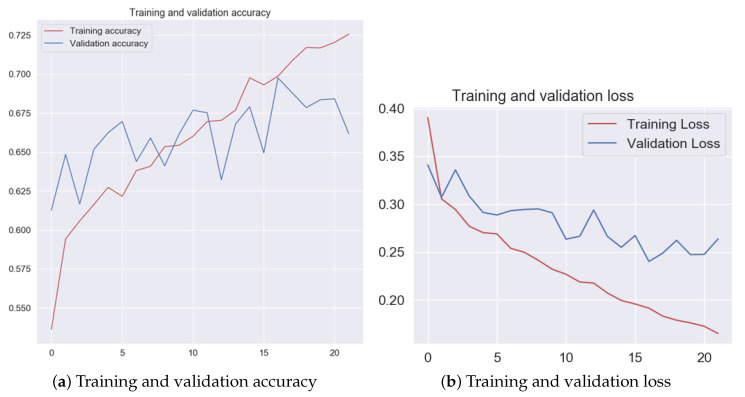
Example function changes for training and validation accuracy (**a**) and loss (**b**).

For each training sequence, one-hundred epochs were used. To optimize the entire solution in terms of the computational efficiency (which is an additional requirement related to the time needed before usable results are obtained; from the manufacturer’s point of view, this time should be as short as possible), an early stopping mechanism was used. The patience parameter was used, set at five epochs, meaning that if during that time, there was no improvement to the solution accuracy, the training process was stopped, and the best obtained model was saved. Figure 7 shows an example of the training process in terms of the accuracy and loss functions.

Since the data used during the experiments were stored in a time series manner (meaning that for each of the tools used, the images of drilled holes were stored exactly in the order they were made), it was additionally incorporated in the overall approach, to try and improve the algorithm’s accuracy. Instead of a single image, sets of consecutive images of different lengths were used for the training process. With such an approach, the algorithm should be able to learn how hole edges change, while the drill is steadily dulling. The window parameter was incorporated into the solution, testing sequences of 5, 10, 15, and 20 images, with no window approach used as the baseline (experts from the furnishing company proposed such window sizes, since they can be incorporated into the production process and used in an actual factory). The first solution, which did not use any window, obtained an overall accuracy of 67% (while the green class recognition had the highest individual scores; see Table 2) and produced a significant number of errors for green-red and red-green misclassifications. Due to the manufacturer requirements, this approach was not satisfactory.

To establish a reasonable benchmark for the current algorithms’ accuracy, different solutions were implemented and tested, with the manufacturer’s requirements in mind. While choosing the algorithms, the main goal was to select as diverse a set as possible, both in terms of the base used (i.e., different pretrained networks and a custom network trained from scratch), as well as the classification methodology (single network or a set of randomly initialized classifiers using the bagging methodology). Since in previous experiments, some of the algorithms were tested and performed well for the overall accuracy parameter, the same algorithms were used for the current comparison. The final set contained the VGG19 pretrained network, 2 ensemble algorithms, the first using 5 and the second using 10 random VGG16 networks, the CNN model trained from scratch, and 5 random initializations of this model. All of those algorithms were trained using the window methodology, starting with no window and finishing at a window size of 20. The accuracy results obtained are presented in Table 3, where each row list the algorithm name.

**Figure 8:**
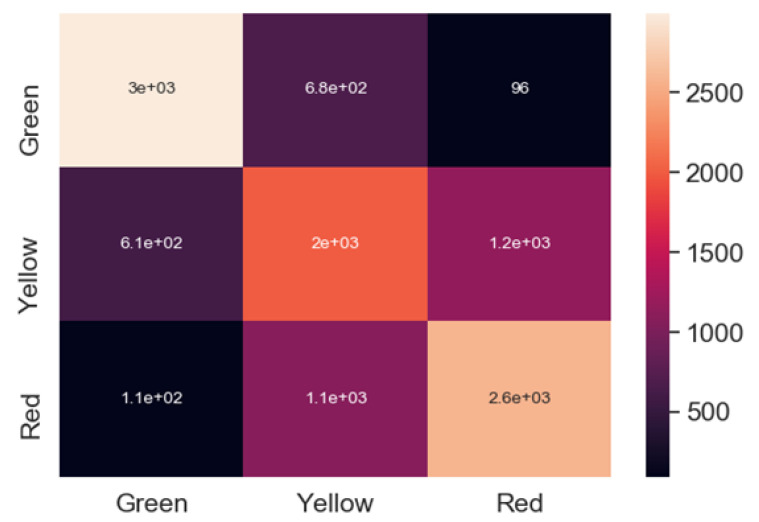
Confusion matrix for the Siamese model with no window used.

**Figure 9:**
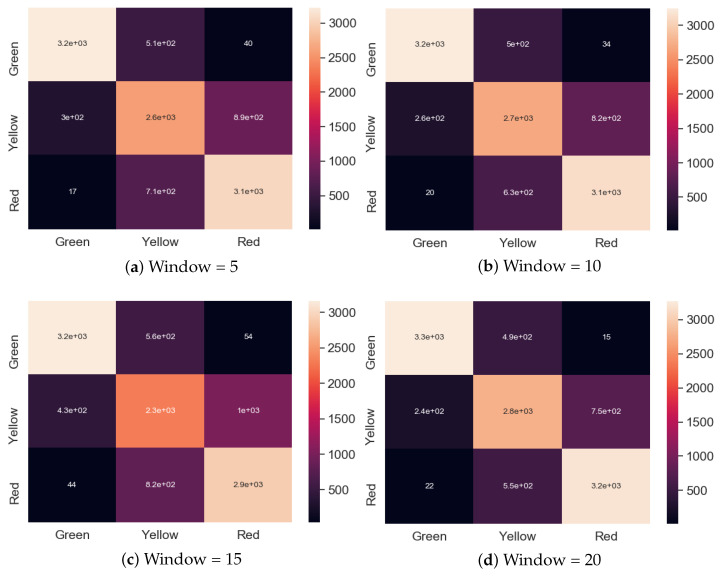
Confusion matrix for the Siamese model with different windows.

Next, the Siamese network approach using different windows was tested, and it achieved the best accuracy results for a window size of 20 (82%). Although for smaller windows, it showed poorer results than some algorithms, it quickly outperformed them for larger windows. While the no window approach produced to many critical errors (see Figure. Figure 8), Figure 9 clearly indicates that increasing the window size also resulted in a better classification rate for the red and green examples, with only 22 red samples classified as green and 15 green samples classified as red. With such a decrease in the misclassification rate, the presented solution reached the benchmark acceptable from the manufacturer’s point of view.

For future work, additional modifications of the presented solution will be considered, focusing on further decreasing the misclassification rate, especially for smaller windows. Furthermore, since storing the data samples in time series, such as in this case, is not always possible, finding some alternative to the windows used in the current approach might also be advisable.

## 6. Conclusions

In this work, the application of a Siamese network to the drill wear recognition problem is presented. Three classes are used for the evaluation: red, green, and yellow. The presented solution uses a custom CNN model and is trained on a considerable data set. Furthermore, the presented solution is evaluated in terms of accuracy, comparing it to different algorithms, implemented with the same manufacturer requirements in mind: reducing the number of misclassifications between the red and green classes. Additionally, since the input images are stored in the exact order in which they were made, the window parameter is introduced, to further increase the classification accuracy. The final solution is able to outperform all tested algorithms in terms of overall accuracy (82% for a window size of 20). Additionally, it is able to accurately distinguish between the red and green classes, with a total number of 37 misclassifications between them (22 red-green and 15 green-red errors). To the best of authors’ knowledge, this is the first application of this methodology to the wood industry. The presented approach is highly adjustable, since in the case of changes in the samples (such as the material used or slight changes to the tools or methods used), transfer learning can be used to retrain the previous model for a new application, without the need to start from scratch.

To summarize, the presented solution achieved an overall accuracy and misclassification rate that fit into the initial acceptable ranges. With the simplified data collection methodology and low initial costs, it is readily applicable to the actual work environment, with very positive, initial feedback from the manufacturer. Furthermore, the Siamese network approach seems very promising, and while further research is still required, it is believed that additional improvement of both the accuracy and critical error rate is still possible.

## Figures and Tables

**Table 1:** Sample counts for each class before and after data augmentation. Values in each cell are presented in the following order: green, yellow, red.

Set Number	Original	Augmented
1	840/420/406	840/840/840
2	840/700/280	840/840/840
3	700/560/420	700/700/700
4	840/560/280	840/840/840
5	560/560/560	560/560/560

**Table 2:** Results for the Siamese network without using a window.

	Precision	Recall	f1-Score
Green	0.81	0.79	0.80
Yellow	0.53	0.53	0.53
Red	0.67	0.68	0.68
Accuracy			0.67

**Table 3:** Classification results for the chosen algorithms, for classification with no window and with different window sizes (5, 10, 15, and 20) for consecutive samples.

Algorithm	No window	W = 5	W = 10	W = 15	W = 20
VGG19	67%	73%	76%	77%	78%
5xVGG16	67%	74%	77%	78%	79%
10xVGG16	67%	73%	76%	77%	78%
CNN-designed	70%	75%	78%	79%	80%
5xCNN-designed	67%	73%	77%	79%	80%
Siamese	67%	74%	78%	80%	82%
